# Perception of misinformation on social media among Chinese college students

**DOI:** 10.3389/fpsyg.2024.1416792

**Published:** 2024-07-04

**Authors:** Bowen Jiang, Desheng Wang

**Affiliations:** School of Journalism and Communication, Shandong University, Jinan, China

**Keywords:** Chinese college students, misinformation, false information, social media, spread of false information

## Abstract

**Background:**

Chinese college students predominantly use social media applications to collect information, communicate with each other, advance their education, and go shopping. Research has highlighted the spread of misinformation or fake news on social media, and this has affected college students negatively as they are the most frequent users of social media.

**Objective:**

This research aims to investigate Chinese college students’ perceptions of misinformation on social media, including their views on the consequences of misinformation, insights into the reasons for its dissemination, how misinformation impacts their mental health, and their perspectives on how to control misinformation.

**Methods:**

This study followed a qualitative approach, selecting 36 participants from 12 universities in China, collecting data through semi-structured interviews, and analyzing the data to enable thematic analysis.

**Results:**

Chinese college students are aware of the adverse impact of spreading misinformation on social media. They believe that false information is disseminated primarily due to inadequate punishment for those who intentionally spread it. Most college students lack proficiency in identifying misinformation, and they expect the government to do more to control the misinformation phenomenon. Moreover, misinformation on social media may cause Chinese college students to feel dysphoric, angry, and even depressed, thereby affecting their mental health. This research indicates that the public and government should make efforts to address the misinformation phenomenon in order to protect college students from being harmed.

## 1 Introduction

Social media refers to internet platforms creating and exchanging content based on user relationships (Dongfang, 2021). It is a general term for social networking websites and communication platforms on which users share insights, experiences, and opinions. It is the most common internet activity among young people (Wartberg et al., 2020).

The main social media sites in China include Weibo, WeChat, QQ, and TikTok. According to the “50th Statistical Report on the Development of China’s Internet” released by the China Internet Network Information Center (CNNIC) (2022), in June 2022, the number of Chinese internet users was 1.051 billion, among which the number of mobile-phone-internet users was 1.048 billion, accounting for 99.7% of the total internet users (China Internet Network Information Center (CNNIC), 2022). Up to 99.3% of mobile internet users have social media apps on their smartphones, and WeChat is the most installed and frequently used social media app in China. WeChat is popular among college students in China. More than 60% of WeChat users are young people (15–29 years old). Since 2020, affected by the COVID-19 epidemic, teaching styles in Chinese universities have changed significantly; many courses and lectures have moved from traditional offline teaching to online teaching. Social media like QQ and WeChat are important tools for Chinese universities conducting online teaching and assignment assistance, thereby increasing the time spent by college students on social media and their dependence on it.

Most Chinese college students use social media, such as WeChat and Weibo, to obtain information. According to Sun and Wei (2023), the proportion of information obtained by Chinese college students through social media accounts for up to 86%, while the use of traditional media platforms, such as television, radio, and newspapers, is relatively low, and the influence of traditional media has gradually diminished in the daily lives of some Chinese college students, while the dominance of digital media has grown significantly. WeChat users check their friends’ circles up to 30–40 times a day, and their primary way of understanding outside information is through social sharing. However, the information they obtain does not necessarily represent the real world (Sun and Wei, 2023). Meanwhile, in choosing social applications, college students have a clear tendency toward community-based platforms, with a WeChat usage rate of 93%, Weibo at 54.9%, QQ Space at 75.8%, and Bilibili at 28.7% (Zhu et al., 2024), demonstrating the importance of social attributes for college students in information acquisition (Wang, 2024). In the social media context, college students position their online identities based on shared interests and hobbies, thereby gaining a sense of belonging.

There are two primary ways to distribute and disseminate social media content: social sharing and algorithm-based sharing (Zhou, 2021). User-generated content is the core concept of social media, the implication being that anyone can create content. Social sharing means that every user can become a content creator and successfully disseminate content through social connections, comments, and forwarding. Information dissemination has shifted from traditional professional editors to the general audience. Everyone becomes a content distributor and content creator. Algorithm-based sharing is based on massive amounts of information on the internet. Specific algorithms determine how content is shared, recommended, or displayed to users. The algorithms consider factors such as content popularity (likes, comments, shares), user interaction history with content, and the sharing behavior of users’ friends or followers (Yang, 2024). The algorithms employed in these platforms are continually updated and refined to better cater to user preferences and behaviors, ensuring that the content delivered remains relevant and engaging. Online platforms use their own algorithms to generate a user profile based on online habits, interests, and hobbies, and recommend content that they believe will be relevant and interesting to users (Zhou, 2021).

With the rise of social media, misinformation is spreading rapidly on the internet (Kim et al., 2021). The development of mobile internet and social media has expanded the speed and scope of information transmission, greatly facilitating people’s information acquisition and exchange, but also bringing about the proliferation of false information. The theory of information manipulation (McCornack, 1992) states that people often manipulate the information they communicate in order to deceive and mislead others, leading to the spread of a large amount of false information. Misinformation is an information activity that misleads the public by deliberately creating or spreading a mix of true and false information, leading to conclusions that are not true (Peng, 2022). It refers to the intentional dissemination of erroneous information and comes in the following forms: false content, misleading intent, and organized action (Yuan, 2022). Terms related to misinformation include false information, disinformation, fake news, and spam, all of which provide false or inaccurate information deliberately created and intentionally or unintentionally propagated (Wu et al., 2019). Network information producers who share information on social media are not professional reporters and have not experienced professional training. The content they produce has not been strictly reviewed by editors, causing false online information to be made public. In December 2017, Chamath Palihapitiya, the former Facebook vice president, stated that Facebook’s social interaction mechanisms, such as “clicking, liking, and forwarding,” did not promote rational dialog or cooperation but instead replicated incorrect information and concealed the truth (Zhou, 2021).

Here are some examples of the type of false information that is typically shared in social media (Sun and Wei, 2023):

- Fake news: This refers to news stories that are fabricated and shared without fact checking. They often contain sensationalized headlines and aim to generate clicks and shares. For example, “BREAKING NEWS: Aliens Found on Mars!”
- Health myths: These are rumors or misinformation about health, nutrition, and medical treatments. They often promise quick fixes or miraculous results, for instance, “Drinking lemon water every morning will cure all your illnesses!”
- Clickbait content: Posts that use sensationalized or misleading headlines to attract clicks, often without providing valuable or accurate information. An example would be, “You Will Never Guess What This Celebrity Did Last Night!”
- Political propaganda: False or misleading information shared to promote a political agenda or candidate. This can include doctored images, false quotes, or distorted facts.
- Chain messages: Posts that encourage users to share a message or action in order to avoid negative consequences, such as bad luck or harm to loved ones. These are usually based on superstition and have no scientific basis.
- Scams and frauds: Offers of products or services that seem too good to be true, often requiring payment or personal information. These aim to defraud users.
- Misinterpreted or outdated information: Content that is taken out of context or is no longer accurate due to changes in events or scientific understanding.

Studies have shown that the speed of misinformation spreading on social media is at least three times that of true information (Sommariva et al., 2018). Users who are repeatedly exposed to misinformation on social media may develop negative beliefs and behaviors and face significant financial and security risks.

The dissemination of false information on various topics has had a negative impact on society and the public. Once false information is accepted, it has a serious negative impact on public perception. Therefore, false information is listed by the World Economic Forum as a major threat to future society (Del Vicario et al., 2016). Existing research focuses on exploring the structural problems of the dissemination of false information, mainly including data mining, recognizing the features of false information content, and understanding the dissemination rules of false information (Zhang et al., 2019; Murayama et al., 2021; Wang C. 2021; Wang W. 2021). In traditional media, the identification of false information was mostly done by journalists through manual verification (Shapiro, 2010). On social media, the way information is disseminated has changed, and the identification of false information has shifted from traditional manual verification to automatic identification. Based on machine learning, image forensics, simulation models, and other methods, supervisors use computers, libraries, and information science to automatically identify and trace the content, expression, and dissemination features of false information (Wang, 2017; Shao, 2018). The anonymous and inexpensive nature of social media forwarding functions facilitates the dissemination of false information, causing false social media information to spread like an infectious disease (Zhang et al., 2019; Wang C. 2021; Wang W. 2021).

Research has shown that social media users play a significant role in the governance of misinformation. According to the “5 W” theory of communication (who says what, in which channel, to whom, with what effect), individuals play roles such as “producer,” “communicator,” and “consumer” in the dissemination of false information (He and Chen, 2012; Bai et al., 2018; Kim et al., 2021). The key to regulating misinformation on social media is to teach users how to identify fake news and not forward it to others (Xiao, 2022). Social media users are affected by multiple factors, including their abilities, social media usage time, education level, age (van der Linden, 2022), information analysis thinking (Pennycook and Rand, 2017), and fact-checking flags (Dongfang, 2021). They often lack confidence and have limited ability to identify, verify, and correct false information (Tandoc et al., 2020; Xiao, 2022). In China, the flood of redundant, false, and misleading internet information has seriously affected the development of society and national stability. Chinese college students’ extensive daily usage of social media apps exposes them to false information and the influence of false information (El Rayess et al., 2018; Li and Zhang, 2018).

The proliferation of misinformation on social media impedes the normal information-seeking behavior of users and poses a significant threat to their health and financial security. As a prominent user demographic, college students frequently engage with and consume a vast amount of information on social media. However, there is a lack of clarity concerning their perception of misinformation on social media and the impact such misinformation has on their mental health and emotional well-being.

This study explores Chinese college students’ daily experiences and perceptions of misinformation on social media from a user’s perspective, including how they perceive false information, how they respond to the dissemination of false information, their ability to distinguish between true and false information, how misinformation affects their emotions, and how to govern false information on social media in the expanding social space.

## 2 Methods

Scholars have undertaken quantitative research using experimental methodologies to assess college students’ abilities to discern false information on social media (Li et al., 2023; Chen et al., 2024). While these findings offer valuable insights, the manner in which college students perceive false information on social media remains unexplored. Therefore, this research does not focus on testing Chinese students’ ability to identify misinformation on social media. Instead, the purpose of this research is to explore what students think of the phenomenon of misinformation on social media based on their experiences and to determine how misinformation impacts their psychological health. Therefore, a qualitative approach was adopted.

### 2.1 Data collection: semi-structured interviews

A semi-structured interview approach was employed to elicit rich data for qualitative analysis. This choice was based on the following considerations: first, the semi-structured design gives the participants ample time and scope to express their diverse views and allows the researcher to react to and follow up on emerging ideas and unfolding events; the findings obtained can be compared among the participants because they are all invited to express their views about the same general themes (Carruthers, 1990). Second, considering that the main aim is to find out how Chinese college students perceive misinformation, semi-structured interviews allow for assessing the participants’ opinions, statements, and convictions, and elicit narratives about their personal experiences (Carruthers, 1990). Creswell (2009) argued that open-ended questions allow the participants to freely voice their experiences and minimize the influence of the researcher’s attitudes and previous findings. Semi-structured interviews enable the interviewer to obtain a clear and comprehensive picture of the subject and gain insights into the participant’s unique experiences. To encourage the participants to speak freely on sensitive personal issues, anonymity was assured. The semi-structured approach also allowed the researcher to control the introduction and flow of the topic.

An interview guide with a list of questions was used to focus the conversation on the research topic (see Table 1).

**Table 1:** The outline of the interviewing questions.

No.	Interviewing questions
1	Can you talk about your perception of social media?
2	What are your thoughts on the negative impacts of false information in social media?
3	How would you rate your ability to identify false information in social media?
4	In your opinion, how capable are Chinese college students, as a group, in identifying false information in social media?
5	Why do you think false information in social media is disseminated so quickly?
6	How does the misinformation affect your emotion?
7	In your mind, how does the phenomenon of misinformation harm the college students’ mental health?
8	In what ways, do you think, should be done to help the college students keep health emotion from the misinformation on social apps?
9	What is your view on the role of the government in combating false information in social media?
10	What do you think are effective measures to control the spread of false information in social media?

### 2.2 Interviewing: online “one-on-one interview” or “group interview”

Online interviewing, as a specialized tool for data collection, offers efficiency, cost-effectiveness, and flexibility (Horrell et al., 2015). It presents new opportunities, improved replication, and enhanced data collection (Cater, 2011; Braun et al., 2017). It also allows flexibility in terms of the time and location of data collection (Cater, 2011; Johnson et al., 2021) and is a highly interactive form of research (Joinson, 2005).

The participants in this study were from various universities and cities. The advantage of conducting online interviews lies in their convenience, time savings, and negligible transportation costs. Furthermore, online interviews offer respondents a comfortable setting, encouraging them to freely articulate their thoughts in a relaxed manner. Therefore, this study employed the Tencent online platform to conduct the interviews.

Initially, the plan envisaged one-on-one interviews, which offered the benefit of a private and undisturbed environment. In such a setting, respondents are more likely to express their views and feelings candidly and directly, thereby providing more accurate information.

However, during the recruitment process, some college students expressed a preference for participating in interviews with their friends. Consequently, a focus-group interview format was also used. The rapid expansion and increased utilization of the internet have fostered new avenues for information collection and dissemination. Many traditional qualitative studies have successfully transitioned to an online format (Murray, 1997; Murray and Sixsmith, 1998; Kozinets, 2010). Researchers select online focus-group interviews in study scenarios involving frequent internet users, geographically dispersed or inaccessible participants, marginalized individuals preferring online interaction, or budget constraints (Bozkurt, 2018). Focus-group interviews are a research technique used to gather data through group discussions on a researcher-determined topic, emphasizing data collection, group interaction, and the researcher’s active role in guiding the discussion (Morgan, 1996). Group interviews differ from focus-group interviews when they are informal, nondirective, or involve unstructured questioning (Frey and Fontana, 1991). Online focus-group interviews involve a group of people, selected according to the purpose of the study, who are willing to share their feelings and opinions about the subject of the study over an online discussion platform (Peacock et al., 2009:119). These interviews involve participants engaging simultaneously with moderators and peers via chat tools, such as online chatrooms, video conferences, or messaging services (Schneider et al., 2002; Reid and Reid, 2005; Fox et al., 2007; Ingram and Steger, 2015; Lijadi and Van Schalkwyk, 2015). Communication occurs rapidly, relying on verbal (oral or written) exchanges (Stewart and Williams, 2005:404–405). Researchers favor this method for its real-time capture of participants’ initial reactions and improvised interactions, resulting in reliable outcomes (Oringderff, 2004:2). Table 2 shows the number of participants in the one-on-one interviews and focus-group interviews.

**Table 2:** Detailed information of the participants.

Category	Item	Number of participants	Category	University	Number of participants
Sex	Male	20	From where	Shandong Normal University	3
	Female	16		Shandong University of Science and Technology	4
Grade year	Grade one	10		Central South University of Forestry and Technology	2
	Grade two	12		Jinan Engineering Vocational and Technical College	3
	Grade three	8		Beijing University of Posts and Telecommunications	2
	Grade four	6		Shandong Xiehe University	5
Interviewing style	One-on-one	20		Changchun University of Science and Technology	2
	Focus-groups	16 (5 groups)		South China University of Technology	2
Users of the top social Apps	WeChat	36		Shandong University of Science and Technology	4
	Tik Tok	34		Shandong University	5
	Little Red Book	32		Northeastern University	2
	Bilibili	32		Shandong Foreign Language Vocational and Technical University	2
Number of total participants: 36			Interviewing in September 2023 and May 2024		

### 2.3 Recruiting participants

Snowball sampling was used to select interviewees. The researcher visited a university, Shandong Xiehe University, in Jinan City and wandered around the campus, chatting with students and telling them about the research objectives. When a student agreed to participate, the researcher recorded their contact information and asked them to recommend friends from other universities who might be interested in this topic. The researcher scheduled interview times with the selected participants.

**Figure 1:**
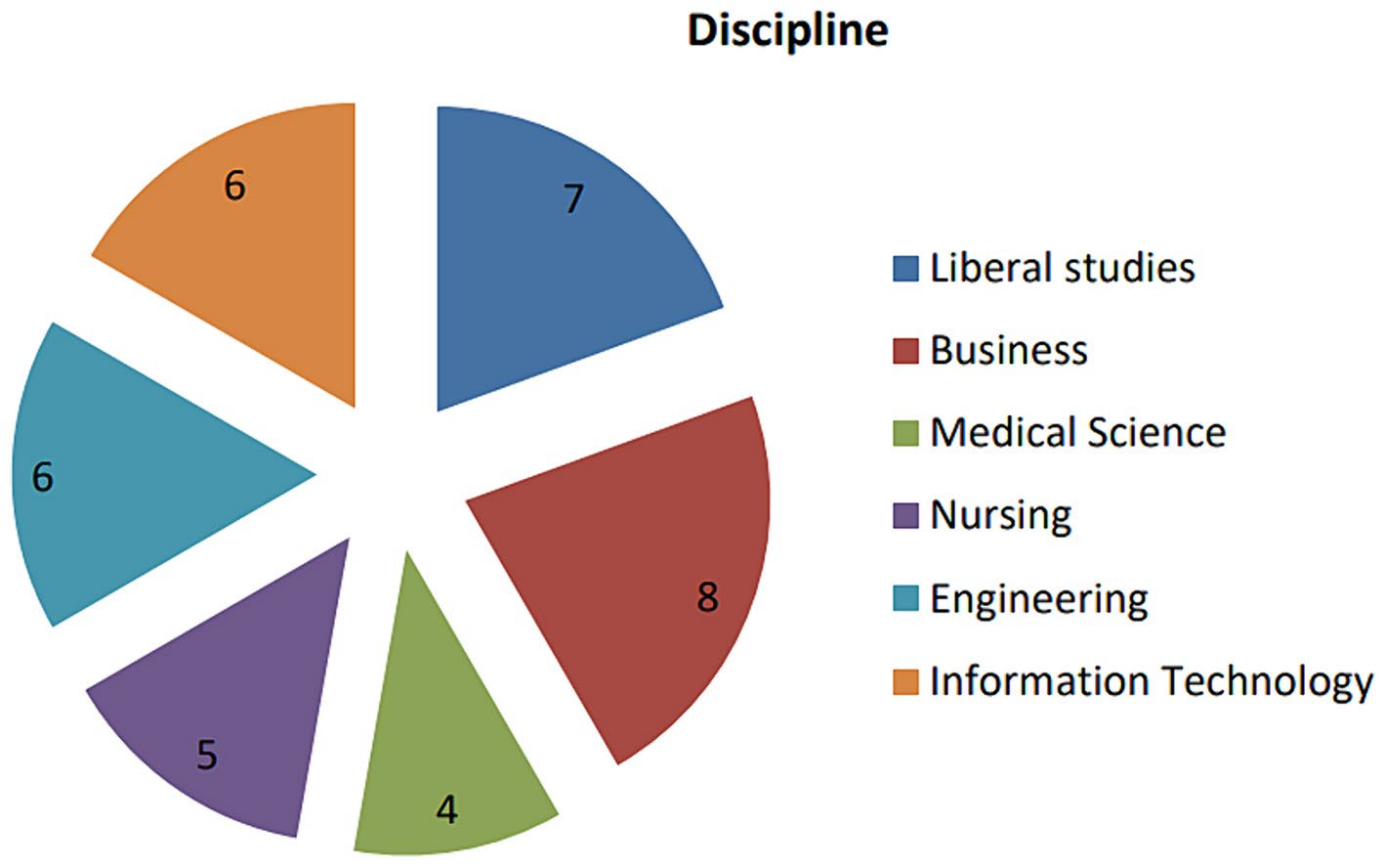
The coverage of the participants’ disciplines.

In terms of participant selection, gender, age, and academic background were considered, and the researcher recruited 36 students to participate in this research. The participants were from 12 universities located in different provinces throughout China, representing a diverse range of educational quality levels among the universities in China. Table 2 displays the participants’ information, and Figure 1 shows the participants’ academic fields.

### 2.4 Data analysis

This study gained the following insights from the data analysis procedure proposed by Ritchie and Spencer (1994): familiarization with the interviews, developing a framework to guide analysis, indexing, charting, and interpreting the data. Based on this, three steps were taken during the data analysis.

The first step was data preparation. The researcher spent several days becoming familiar with and immersed in the data by listening to the recorded interviews, reviewing notes, and transcribing them.

Interview data were recorded (with participants’ consent) using a voice recorder and subsequently transcribed. Conversations that were not recorded were reconstructed based on notes taken during the interviews.

The second step involved coding the transcripts. Ritchie and Spencer’s (1994) framework method was used to create a thematic framework. This study employed three rounds of coding. In the first round, a concept-based coding approach was used to identify information relevant to the research questions. All codes related to the research questions were identified during this round. Although theory-driven coding was primarily used, the researcher also employed data-driven coding to capture emerging themes. The second round of coding involved analyzing the data to identify patterns related to social media misinformation, going beyond the research questions to facilitate deeper discussion. In the third round, the categories and themes developed in the first two rounds were compared, and the data were reviewed to check for consistency and to re-code any contradictory data.

The third step involved checking the consistency and accuracy of the coding results. At this stage, two of the researcher’s colleagues with extensive experience in R&D were asked to verify the consistency, accuracy, and saturation of the data coding results.

- Consistency

In order to keep the results accurate and consistent, we took the following steps:

First, random sampling inspection: We randomly selected 10% of the encoded data for review to ensure that the coding results aligned with the established coding standards.

Second, cross-validation: We assigned the same data to different coding personnel or teams for coding. We compared their coding results, identified any inconsistencies, and discussed and, if necessary, corrected them.

- Research ethics

Regarding ethical considerations, we adhered to the following procedures:

Privacy protection: Prior to the commencement of the interviews, we clearly informed students about how their information, including interview recordings, transcriptions, and data analysis, would be processed and stored. We ensured the secure storage of all interview materials (recordings, transcripts, and reports) to prevent unauthorized access. The students gave their consent for the possible publication of their views. They were assured that their anonymity would be preserved. In scenarios where the interview results could potentially have a negative impact on students’ rights and interests, we took appropriate measures before making the findings public. For instance, during participation and transcription, we replaced the participants’ names with identifiers, such as “P,” followed by a number (e.g., P1 to P36).

Informed consent: Prior to the commencement of the interviews, we gave the students a detailed informed consent form outlining the purpose, methodology, potential risks, and benefits of the study. We insured that the students had ample time to read and comprehend the informed consent form and that they signed it without coercion or inducements.

Voluntary participation: We emphasized that participation in interviews was voluntary and that students could opt out at any time without any adverse consequences.

Non-inducing questioning: We refrained from using suggestive or leading questioning techniques, thereby ensuring that students’ responses were based solely on their own experiences and perceptions.

## 3 Results and discussion

**Figure 2:**
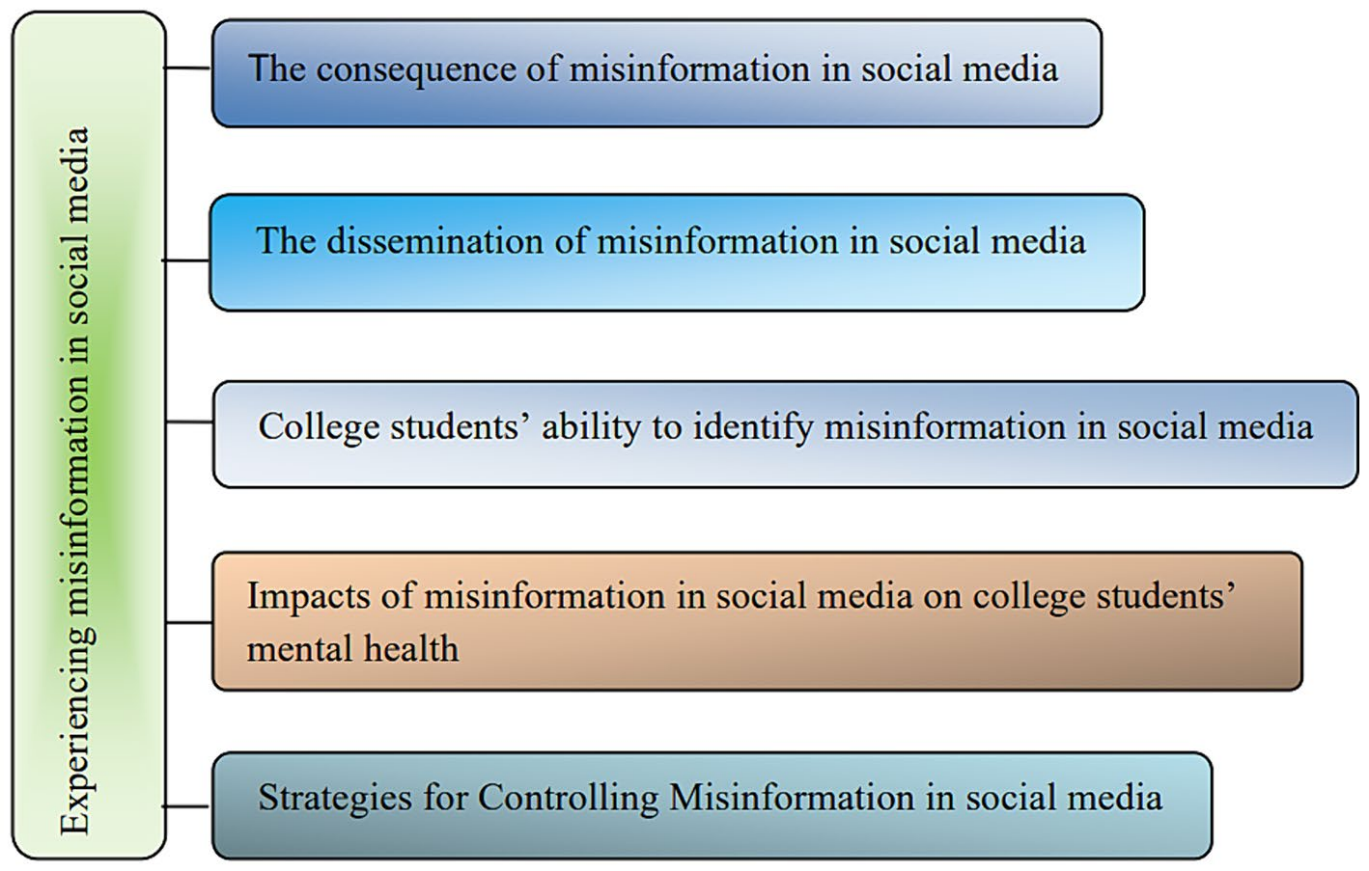
Structure for analyzing the Chinese college students’ experience the misinformation in social media.

The data analysis process was based on Ritchie and Spencer (1994) thematic framework method. The structure for analyzing the data is shown in Figure 2. Data were coded, and the following five themes were identified: what the college students think are the consequences for society of misinformation in social media, why and how false information is spreading, what the college students think of their ability to recognize misinformation, what the impact of the phenomenon of misinformation is on college students’ mental health, and what strategies the college students think should be implemented to control the spread of misinformation on social media.

### 3.1 Consequences of false information on social media

#### 3.1.1 Harm to social stability

The widespread dissemination of online rumors in the social media environment can cause social unrest and exacerbate public panic. With increasing internet usage rates and the growing number of internet users in China, some participants believe online rumors could cause severe harm to social stability.

“.. you know, with COVID-19 in 2020, so much fake news, we rushed for a particular medicine one day, and hunted for another later, but such information spreading on social media was not true, which just made the public worried all the time, and made them do stupid things..” (P20, interview, Sept., 2023).

The circulation of rumors among a large group will also intensify public anxiety. As P16 said,

“People around me are scared by hearing such rumors, and most of them cannot focus on their work..” (Interview, Sept., 2023).

Distorting facts based on real-world events and sharing them online is irresponsible speech that can easily trigger social unease, mislead citizens, and provoke social conflicts, leading to panic.

“The dissemination of false news among individuals can exacerbate social conflicts, leading to widespread panic and eroded trust in the government, especially when rumors pertaining to public health circulate.” (P13, interview, Sept., 2023).

Research shows that the characteristics of online rumors make them difficult for ordinary people to detect, and their widespread dissemination poses a serious threat to family harmony, social stability, and national prosperity (Xiao, 2022). Currently, people are exposed to the internet at a younger age, and some teenagers have limited ability to distinguish the truth of online information. They are easily influenced by online rumors, potentially harming themselves and those around them. Allowing network rumors to spread freely on the internet seriously interferes with the stability and orderly development of society as a whole (Zhou, 2021).

#### 3.1.2 Damage to social trustworthiness

The online world, like the real world, requires integrity. The lack of internet credibility may lead to the circulation of fake news, causing significant damage to the real world.

“Some lawbreakers exploit people’s curiosity and maliciously fabricate false information on the internet, ranging from slandering individuals to distorting national policies.” (P26, interview, Sept., 2023).

The abundant fake information on the internet has seriously affected people’s cognitive abilities and caused significant social harm. Some network rumors in social media environments cater to people’s psychological needs, circulating widely and causing social conflicts and trust crises.

As P15 said,

“Currently, China is in a crucial period of reform, with economic operation methods and social development status facing transformation pressure. During this stage, various social conflicts converge, often becoming the focus of online discussions.” (Interview, Sept., 2023).

In this sensitive period, the public’s psychological resilience is extremely fragile and vulnerable to adverse information (Peng, 2022). Network rumor creators use public uncertainty or depressed psychology during social transformation periods to intentionally fabricate and spread false invitations to attend public events, thereby misleading citizens (Shao, 2018). These messages are directly presented to the public through modern online social media, causing ignorant internet users to attend collective social events, posing a significant threat to social security and public safety. Therefore, social media network rumors can be considered to provoke social unrest and trust crises.

### 3.2 The spread of false information on the internet

#### 3.2.1 Low-cost dissemination of false information

Most college students think that the cost of spreading fake information is low. The reason-based approach of the print media era has been replaced by the emotional participation of internet users of social media networks in dissemination activities.

“These users often do not actively analyze the authenticity of information objectively before spreading it. When confronted with doubts from others, they can simply delete the information to extricate themselves from the situation and quickly move on.” (P19, interview, Sept., 2023).

This zero-cost dissemination makes it difficult for social media platforms to cultivate good user behavior and enable users to establish reasonable cognition at an ideological level (Zhou, 2021). Over time, this inevitably leads to a deterioration of the information dissemination environment on social media platforms.

#### 3.2.2 Unrestricted spread among acquaintances

Young people usually believe information passed on by friends. “When I see the news from my friend’s social account, I seldom doubt it, and just believe it,” said P1 (Interview, Sept., 2023), and the majority of the participants expressed the same idea.

Based on Maslow’s hierarchy of needs theory, people have a need for social recognition, often originating from their “acquaintances” and linked to interpersonal relationships (Peng, 2022). This can result in chain-like sharing that spreads from one person to a wider network, thus expanding its influence. While not every share is voluntary or intended to be presented to “acquaintances,” social pressure can compel people to share information. Li and Zhang (2018) proposed that acquaintances’ share acts as a chain transmission, and that when the source of information is incorrect, there can be a widespread dissemination of false information. From a psychological perspective, people in this context often have a strong sense of mutual identification and may neglect the issue of information authenticity during dissemination. Acquaintances’ sharing has become one of the reasons for the high occurrence of false information in the social media era (Wang, 2024).

#### 3.2.3 Influence of sharers

Young people browsing information on social media are amenable to the influence of people they admire.

“If the star, such as a singer or a football player, who I like, says something, or shares some information, I would absolutely follow him/her, and I would also share such news on my social media.” (P15, interview, Sept., 2023).

In the case of news-related false information, the direct source is typically news agencies and websites, while the indirect source is often institutions, websites, or individuals that republish the information (Dongfang, 2021). The direct source of information on social media is the original creator of the content (institution, website, or individual), and the indirect source is the sharer, the user who shares the information. Social media users tend to trust and are likelier to share news or information that is shared by trusted public figures. They are more inclined to like, comment on, and forward information and viewpoints that they believe are true and aligned with their own beliefs (Wang, 2021). Opinion leaders play a crucial role in the information dissemination chain on social media platforms. They can gain attention and trust from relevant groups and exert significant influence on the spread of information. Opinion leaders with a large following can accelerate the dissemination of rumors, and users’ familiarity with and trust in these leaders can influence user behavior (Bai et al., 2018).

#### 3.2.4 Disregard for information sources

Some college students said that they do not like to check the information source.

“I do not have it in my mind, I mean, I do not particularly care about the information source every time while going through the news.” (P2, interview, Sept., 2023).

In the traditional media era, whether it was newspapers or television, media outlets had distinct labels and characteristics, with some focusing on serious news, others on entertainment gossip, and others on public and commercial programming. Audiences typically select media content that matches their tastes and preferences based on the media’s positioning (Wang, 2017). In the past, audience members took an active role in obtaining information and choosing which newspapers or television programs to consume. However, in the era of online media, digital platforms have broken the boundaries of user contact information. Currently, the massive amount of information, personalized push notifications, and 24-h updates provide internet users with information through social media friends, causing them to follow others’ shares, rather than visit specialized news websites (Shao, 2018). Therefore, for ordinary social media users, the content of the information or the channel from which they obtain it is often more important than the source of the information. Peng (2022) study showed that many people can clearly remember the content of a news story but less than 50% can remember the name of the news publisher. Users are more concerned about “trending topics” and “popular posts in social media circles” than about the information source (Wang, 2021). Whether the information is true or not is not an initial concern for most social media users.

### 3.3 Identifying false information on the internet

If we discover that information is false, we will not be misled. The college students talked about how to identify misinformation. However, most of them are not optimistic about identifying false news.

#### 3.3.1 Poor ability of college students to identify misinformation

The college students think they are not capable of identifying misinformation on social media.

“I am not confident in myself, you know, I am often misled by fake news,” said P19 (Interview, Sept., 2023), and the majority of the participants expressed similar views while talking about college students’ ability to identify misinformation.

Due to a lack of confidence in verifying and correcting fake information, teenagers are easily influenced by fake information on social media, leading to a widespread problem of distinguishing between fake and real information.

“In my mind, I think college students have a limited ability to distinguish fake information and tend to rely on external information sources for verification.” (P14, interview, Sept., 2023).

Although college students recognize the importance of identifying fake information on social media and have a high awareness of its significance, their discrimination accuracy is not high, especially in the comprehensive application of traceability and cross-validation (Li et al., 2023). People aged 35–45 are the most skilled age group at recognizing misinformation (Zhu et al., 2024). The subject of the information is related to the level of discrimination awareness and ability, and Song (2018) proposed that the college students show higher discrimination awareness and accuracy in public health information compared to social news (Song, 2018). Li et al. (2023) and Chen et al. (2024) support the view that Chinese college students are good at identifying misinformation.

#### 3.3.2 College students tend to have a relatively casual attitude toward distinguishing fake information

College students’ behavior in screening fake information in the social media environment exhibits noticeable individual characteristics.

“Sometimes, I may think this news is suspicious, but I do not have time to seek confirmation.” (P10, interview, Sept., 2023).

“I seldom spend time verifying the information. It’s a time-consuming process.” (P7, interview, Sept., 2023).

The present research study shows that to minimize costs and effort, college students often rely on their personal knowledge reserves when screening for fake information. They rarely use comprehensive screening behaviors, such as traceability and cross-validation. It is only when they cannot determine authenticity based on their personal knowledge that some college students implement diversified screening strategies. According to Xiao (2022), young people generally perceive the traceability or verification of information retrieved through search engines or social media as accurate.

### 3.4 Impacts on college student’s mental health

Concerns and pressure stemming from misinformation on social media can significantly impact the mental health of college students.

“Misinformation on social media is frequently coupled with exaggerated and misleading content, which readily trigger feelings of anxiety and depression in me.” (P33, interview, May 2024).

College students may feel uneasy due to doubts about the authenticity of misinformation, or become confused and frustrated because of their inability to distinguish truth from falsehood.

“Misinformation on social media often portrays an idealized and unrealistic portrayal of life. I often feel inferior because I compare myself unfavorably to others.” (P27, interview, May 2024).

Moreover, misinformation may prompt college students to rely excessively on social media for interpersonal relationships, thereby neglecting real-world social interactions. This overreliance can foster social barriers, leading to feelings of loneliness and isolation.

The dissemination of misinformation can erode college students’ trust in information. “Due to the prevalence of misinformation, I sometimes adopt a skeptical stance toward all social media content, even fostering a sense of distrust toward real-world information.” (P33, interview, May 2024).

This trust crisis may hinder college students’ decision-making abilities or foster excessive vigilance toward others.

### 3.5 Strategies for controlling misinformation on social media

Participants shared their thoughts on how to control misinformation.

#### 3.5.1 Enhancing college students’ information literacy

Most participants said that college students’ computer literacy must be improved as a means of addressing social media information misinformation.

“In the past, in pre-internet days, fake news was not so easy to spread, and people did not get today’s flood of misinformation. I know most of us do not have the ability to identify misinformation. That’s why I think it’s necessary to provide some training for college students to improve our literacy to recognize the fake news in our daily life.” (P3, interview, Sept., 2023).

Yuan (2022) points out that improving college students’ information literacy is a long-term task closely linked to social development. While combating false information, audiences should assume a degree of responsibility, rather than relying solely on technology and the media to address the problem.

“With training, we would have the necessary knowledge to identify misinformation. We would like to be aware while reading misinformation and to doubt its veracity.” (P23, interview, Sept., 2023).

In the “magic bullet theory,” meaning that media can penetrate people’s brains and quickly create effects, users are no longer passive recipients (Li et al., 2023). Therefore, current audiences or social media users should cultivate independent thinking, possess the ability to discern truth from falsehood, overcome unhealthy media habits, and establish proper communication awareness. Enhancing the media literacy of the public and encouraging active participation from social media users is crucial in combating false news.

Media and information literacy education should be a starting point toward cultivating the effective ability to identify fake information. Thoughtful and targeted actions are particularly crucial (Song, 2018). According to the Fogg model (B = MAT), behavior occurs when there is motivation, ability, and a trigger. Behavior = ability × motivation × trigger (Foog, 2021). Ability is a significant factor influencing individual behavior. Therefore, improving the ability to identify fake information is essential in reducing the likelihood of the secondary dissemination of false information on social media. Currently, media and information literacy is widely recognized as the most effective measure to tackle fake information, encompassing media literacy, information literacy, and digital literacy (UNESCO, 2022). Among these, information identification is the core of media and information literacy (Grizzle et al., 2013). It is vital to cultivate students’ critical thinking skills, “the ability to critically analyze obtained information,” and enhance their ability to distinguish and reason out the authenticity of information and the intentions behind its dissemination. This process will address the issue of college students’ reliance on external verification sources during multi-source cross-validation and their lack of ability to evaluate the authenticity, authority, and reliability of such sources. Simultaneously, their immunity, discrimination, and response to social media fake information should be strengthened.

#### 3.5.2 Reducing the cost of dealing with false information through technological measures

The anonymity of users on social platforms makes it challenging to trace the source of information. This has always been a difficulty in combating fake information.

“Although I know I should treat the information seriously, especially when I think it is probably not true, it annoys me to have to make efforts to verify the information.” (P18, interview, Sept., 2023).

Blockchain technology, with its characteristics of decentralization, immutability, and traceability, is expected to become an important tool for information regulation in the future (Song, 2018). However, its limitation lies in its inability to predict the dissemination of fake information.

Some students mentioned big data as potentially helping the public to identify misinformation. P7 said, “Big data analysis technology can also be a powerful tool in combating fake information” (Interview, Sept., 2023). Big data online analysis technology can effectively play the role of a watchdog for the vast amount of information on social media.

This research found that college students use relatively simple behavior strategies when identifying fake information on social media, primarily due to their tendency to follow the principle of minimum cost and effort and their lack of awareness of auxiliary means and measures for identifying fake information. To some extent, this has also affected their ability to identify fake information. Currently, some countries employ “horizontal reading” training to assist in identifying fake information (Wineburg et al., 2016). This method has been adopted and implemented by professional fact-checkers and the Stanford History Education Group. In China, dedicated websites have been established to help the public verify fake information. They include the “China Internet Joint Dissemination Platform,” Tencent News’ “Fact-checking Verification Platform,” “WeChat Dissemination Assistant,” and Sina Weibo’s “National Dissemination Platform.” Furthermore, given the varying susceptibility of people to fake information, identifying individuals with high susceptibility factors can aid in developing psychological prevention strategies to reduce the subsequent cost of identifying fake information (Cao and Ke, 2023).

#### 3.5.3 The government should ensure transparent and timely dissemination of information

The college students proposed that the government should play a core role in governing the misinformation phenomenon in social media.

“First, the government is expected to achieve transparent and timely publication of important information, enhancing its ability to refute rumors. We know that proving the falsity of online rumors takes time, and only when widely spread online rumors are proven false can the network of rumors be cleared and the online environment purified.” (P11, interview, Sept., 2023).

In practice, the verification process for the authenticity of network information is relatively slow, and various types of information flood cyberspace, including various types of online rumors (He and Chen, 2012). Therefore, although it is impossible to eliminate the time difference, it should be recognized that the faster the truth of opaque online information can be made public, the more effective it will be in preventing the generation and dissemination of online rumors.

Online rumors can be clarified promptly through comprehensive cooperation among all sectors of society. The identification and governance of false information have become an important issue (van der Linden, 2022). From the government’s perspective, this means that there is a need to improve the processing system for adverse information and the press conference system. There is a need for timely publication of information that the public is eager to know through official government websites and official Weibo platforms, effectively safeguarding the public’s right to know.

#### 3.5.4 Strengthening internet legislation

The college students mentioned that laws related to misinformation should be enacted as early as possible.

“It’s a new environment where misinformation is common, and no specific regulations in the law tell people to do the right thing while creating content online.” (P11, interview, Sept., 2023).

In the face of various issues arising from internet development, it is crucial to reinforce internet legislation. Ignoring the various acts of spreading rumors and lies on the internet will inevitably lead to more people joining the ranks of rumor creators and spreaders (Yuan, 2022). This becomes an alarming phenomenon. Therefore, given the current situation of a network society where network rumors are constantly emerging, it is imperative to establish and improve the laws and legislative work of the network society, further regulating the behavior of information dissemination by social media on the internet (Zhou, 2021). Network social media platforms also need to enhance self-regulation and establish relevant industry laws and supervisory regulations, thereby promoting social media platforms to operate more maturely, professionally, and legally.

#### 3.5.5 Measures to protect the college students’ mental health

“Our university should intensify efforts to counter the spread of false information online and foster students’ awareness of prevention. Furthermore, we can leverage social media to deliver mental health education to college students and boost their mental well-being awareness.” (P11, interview, May, 2024).

Several students also emphasized the importance of enhancing media literacy education and refining college students’ abilities to discern and evaluate false information (P27, P29, P30, P35, and P36). When gathering information, it is recommended that college students prioritize information from reputable media outlets and institutions to minimize the risk of encountering false information. The healthy and rational utilization of social media should be promoted, and overreliance on social media for building and sustaining interpersonal connections should be discouraged.

## 4 Conclusion

Social media serves as a vital avenue for Chinese college students to stay informed about current events. Although college students possess some awareness of misinformation on social media, their ability to identify it requires improvement. The spread of misinformation on social media remains a pressing challenge among college students, one that needs to be addressed.

This research indicates that college students are aware of the consequences of the misinformation phenomenon, but they are not skilled at identifying misinformation, and they do not find it easy to verify the information on social media to ensure a better information environment. Moreover, the phenomenon of misinformation is harmful to college students’ mental health, making them angry and causing them to mistrust the world around them. College students expect the government to do more to fight the misinformation phenomenon, and, simultaneously, to enhance college students’ literacy by offering training classes in universities on strategies to identify misinformation.

A limitation of this study lies in the relatively small sample size of 36 participants. Furthermore, the data collection pertaining to psychological impacts was insufficiently profound and requires further refinement. In our future research, we intend to continue to explore the impact of social media on college students by using a multifaceted approach encompassing experimental methods, questionnaire surveys, and interviews.

## Data availability statement

The datasets presented in this article are not readily available because This refers only to raw, anonymized data. Never share participant’s identifiable data. Requests to access the datasets should be directed to BJ, 202120504@mail.sdu.edu.cn.

## Ethics statement

This study was approved by the Ethics Committee of Shandong University. Informed consent was provided for all participants. Written informed consent from the [patients/ participants OR patients/participants legal guardian/next of kin] was not required to participate in this study in accordance with the national legislation and the institutional requirements.

## Author contributions

BJ: Writing – review & editing, Writing – original draft, Visualization, Software, Resources, Project administration, Investigation, Funding acquisition, Formal analysis, Data curation, Conceptualization. DW: Writing – review & editing, Validation, Supervision, Methodology.
